# Public Health, Nutritional Behavior, and Nutritional Status: An Editorial Synthesis and Roadmap for Precision Nutrition

**DOI:** 10.3390/nu17182968

**Published:** 2025-09-16

**Authors:** Sabina Lachowicz-Wiśniewska, Agata Kotowska

**Affiliations:** 1 Department of Medical and Health Science, University of Kalisz, W. Bogusławskiego 2 Square, 62-800 Kalisz, Poland; 2 Department of Biotechnology and Food Analysis, Wroclaw University of Economics and Business, Komandorska 118/120 Street, 53-345 Wroclaw, Poland; 3 Institute of Sociology, University of Rzeszów, Rejtana 16C, 35-959 Rzeszów, Poland

## 1. Introduction

In the face of growing lifestyle-related health concerns across many populations—such as obesity and poor diet—understanding the determinants of these behaviors is crucial for designing effective public health strategies. Nutrition exerts a fundamental influence on both physical and mental health, and health-promoting dietary modification is not only relatively straightforward but also an extremely effective means of preventing and treating many serious diseases and extending life.

The theme of this Special Issue of *Nutrients*, “Public Health, Nutritional Behavior and Nutritional Status—2nd Edition,” lies at the intersection of public health, dietary behaviors, and nutritional status, with an emphasis on population-level solutions (social policies, education, and the food environment), as well as tools for precision nutrition and mHealth. Framed this way, the agenda addresses a dual contemporary challenge: on the one hand, the high prevalence of diet-related diseases; on the other, persistent undernutrition and health inequities.

In this context, data from Poland provide a valuable reference point, revealing a gap between young people’s high self-declared awareness and their actual habits (insufficient physical activity, low fruit and vegetable intake, frequent emotional eating, and infrequent label reading). Social media plays a prominent role in shaping beliefs. This picture underscores the need for system-level actions and projects that support behavior change within everyday living ecosystems. In parallel, research on nutrition personalization and digital support (e.g., i-Diet within Stance4Health) shows that accurately reflecting real-world choices (including portion sizes) and embedding modules that mirror local habits and preferences can enhance adherence to recommendations. Thus, precision nutrition becomes a practical bridge between population policies and individual decision-making.

This synthesis highlights key findings from the empirical studies and reviews included in the Special Issue. The topics focus primarily on the environment and public health policies; nutrition in children and adolescents (snacks, fats, portion size); dietary behaviors and patterns in relation to health risk; biological age and risk markers; personalization and digital tools for health promotion; and the burden on—and inequities in access to—the healthcare system.

## 2. Results

A study among Polish school and university students revealed a marked knowledge–behavior gap. Most participants accurately described healthy eating and reported adhering to dietary guidelines; however, despite their professed health consciousness and familiarity with recommendations, 43% engaged in emotional eating. Fruit and vegetable intake fell below recommended levels, and nearly half of the respondents were insufficiently physically active. Only 36% reported routinely reading food labels, and many selected products containing additives. Appearance-related stress was common (52%), alongside widespread sleep insufficiency and inadequate physical activity. Given the rapid rise in overweight and obesity among Polish children and adolescents, there is an urgent need for broad, state-level social policy measures, including environmental interventions (schools, universities) and more effective use of digital channels for health communication [contribution 1].

Excess caloric intake is the principal driver of obesity. Unhealthy dietary habits—such as increased eating frequency, larger portion sizes, and high snack consumption—contribute to its development. To test whether differences in snack perception across BMI categories might underlie obesity risk, investigators assessed the perceived energy content and portion size of snacks. BMI appeared not to influence these perceptions; instead, the study revealed generally low literacy in caloric assessment. Most individuals misestimate snack energy content, which may contribute to obesity development [contribution 2].

An analysis of sweets and savory snack consumption among children and adolescents showed that these foods are the main contributors to excess total fat and to saturated (SFA) and trans fatty acid (TFA) intakes. In a cross-sectional sample of Polish pupils aged 10–15 years, mean fat intake from snacks was 34.5 g/day—covering nearly 47% of the recommended daily intake. Over 12% exceeded recommendations for total fat, 20% surpassed SFA limits, and more than 30% crossed the TFA threshold. The highest fat intakes were observed among 10-year-olds, of whom over 60% exceeded the upper level for TFA intake; intake was also highest among children with overweight or obesity. Snacks are therefore a principal source of unhealthy fat intake in school-age children—especially the youngest and those with excess body weight—underscoring the need for policies limiting TFAs, precise portion size education, and reform of the school food environment to increase the availability of healthier options [contribution 3].

In a large cohort of South Korean adults, significant associations were identified between consumption of food groups and the risk of self-reported stroke and myocardial infarction. Pattern-based analyses (rather than single-food approaches) more accurately captured risk profiles and can inform primary prevention. Diets rich in protein and fruit may confer protection, whereas high-calorie patterns dominated by rice and processed red meat, as well as excessive coffee intake, may increase the risk of stroke or myocardial infarction [contribution 4].

Based on the premise that indices extending beyond conventional BMI better capture early metabolic disturbances, investigators evaluated the utility of “metabolic age” (MA) for predicting insulin resistance and cardiometabolic risk. The evidence indicates that MA—while communicatively appealing—has limited predictive value when used in isolation; its usefulness increases only when combined with a biochemical panel (glycaemia, lipids, insulin resistance indices) and with an assessment of fat distribution (visceral vs. subcutaneous adiposity). Thus, biological “age” should be incorporated as one component of an expanded, multiparametric risk assessment, rather than being used as a substitute for it [contribution 5].

In the Stance4Health study on dietary behaviors and portion size selection among 224 participants in Spain and Germany, cross-country differences were confirmed: Spaniards tended to choose larger portions of rice, meat, and legumes, whereas Germans more often selected larger portions of stews, lasagne, and pizza; adherence to the Mediterranean diet also differed, alongside small but significant differences in BMI. These data were generated using the i-Diet app’s portion size module (XXS–XL with a photographic atlas), flexible meal composition (bread, beverages, desserts), and allergy/intolerance management, with the aim of improving adherence and durability of change. The findings can be applied to more precisely tailor personalized nutrition applications to diverse user needs [contribution 6].

Drawing on data from 1703 adults in Singapore’s Population Health Index, adult undernutrition was associated with increased emergency department use, hospitalizations, and costs, underscoring the need for policies to counter undernutrition in parallel with obesity control (screening, nutritional support, identification of high-risk groups). Undernutrition emerged as a critical yet modifiable risk factor for preventable hospitalizations [contribution 7].

A study of Spanish workers examined links between sociodemographic factors, lifestyle behaviors, and vascular age (VA) and heart age (HA). Aging of the vascular and cardiac systems depended on chronological age, sex (with accelerated aging in postmenopausal women), and socioeconomic status. Physical activity and adherence to the Mediterranean diet were associated with lower VA and HA, whereas excessive alcohol intake, smoking, and low physical activity accelerated aging. Many of the determinants of VA and HA are therefore modifiable, offering actionable targets for healthcare practice [contribution 8].

Among aging adults in Canada (Canadian Longitudinal Study on Aging, CLSA), dietary behaviors were related to social participation. Persistent social isolation affected changes in fruit and vegetable intake among women only, whereas limited diversity of social participation influenced fruit and vegetable intake in both sexes. These findings justify further longitudinal research into the complex interplay between social engagement and dietary behaviors [contribution 9].

## 3. Conclusions

Taken together, the contributions in this Special Issue highlight a consistent pattern: knowledge alone is insufficient to shift behavior, and durable change requires supportive environments, clear standards, and person-centered tools. First, policy and food environment levers—especially TFA elimination/reduction, reformulation, and healthier school settings—are pivotal where snack-derived fat and TFA exposure is high. Second, precision nutrition can operationalize guidance by mirroring lived choices (e.g., portion size modules, allergy/intolerance management) and adapting them to cultural contexts, thereby improving adherence and equity of access. Third, routine screening and integrated care should address both overnutrition and undernutrition, using multiparametric risk panels (biochemistry + body fat distribution) rather than single surrogate markers (e.g., metabolic age alone). Finally, future research should prioritize longitudinal and quasi-experimental designs, harmonized outcomes, FAIR/open data, and the integration of social determinants and mental health correlates to close the knowledge–behavior gap at scale. These recommendations form a coherent agenda for linking population health policy with individualized nutrition across the life course.

